# Assessment of Two Commercial Serological Assays for the Diagnosis and Post-Treatment Follow-Up of Strongyloidiasis in a Cohort of Patients with Chagas Disease

**DOI:** 10.3390/pathogens15060627

**Published:** 2026-06-12

**Authors:** Luis Gil-Gallardo, Marina Simon Páez, Javier Nieto Martínez, Maria Delmans Flores-Chavez, María Asunción Iborra-Bendicho, Manuel Segovia-Hernández

**Affiliations:** 1 Tropical Medicine Unit, Department of Microbiology, Hospital Clínico Universitario Virgen de la Arrixaca, 30120 Murcia, Spain; luisgilga@gmail.com (L.G.-G.); marina.simon.paez@gmail.com (M.S.P.); msegovia@um.es (M.S.-H.); 2 Murcia Biomedical Research Institute (IMIB-Arrixaca), 30120 Murcia, Spain; 3 Department of Genetics and Microbiology, University of Murcia, 30100 Murcia, Spain; 4 Reference and Research Laboratory on Parasitology, National Centre for Microbiology, Instituto de Salud Carlos III, 28029 Madrid, Spain; fjnieto@isciii.es

**Keywords:** strongyloidiasis, serological screening, sensitivity, specificity, ELISA, treatment follow-up

## Abstract

Strongyloidiasis is a neglected tropical disease that frequently coexists with Chagas disease (CD) among individuals from Latin America. Serology is increasingly used for the diagnosis and post-treatment follow-up of strongyloidiasis; however, comparative data on commercially available assays remain limited. We conducted a cross-sectional evaluation in a cohort of fifty-five patients with previously treated CD attending a Tropical Medicine Unit in Spain. All patients underwent serological screening for *Strongyloides stercoralis* using two commercial ELISA assays: the *Strongyloides* IgG ELISA kit (DRG Instruments GmbH) (DRG) and Anti-*Strongyloides* ELISA IgG (Euroimmun Medizinische Labordiagnostika AG) (Euroimmun). A composite reference standard defined infection as seropositivity in combination with eosinophil count (>0.5 × 10^3^ eosinophils per µL) or seropositivity in both assays. Infected individuals received ivermectin and were reassessed one-year post-treatment. Thirty-seven patients (67.3%) met the criteria for strongyloidiasis. Concordance between the assays was substantial (κ = 0.80). According to the composite study definition, both assays identified most patients as infected. At one-year follow-up, significant reductions were observed in DRG and Euroimmun indexes, as well as in eosinophil count (all *p* < 0.001). Treatment response rates were 83.3% with DRG and 73.3% with Euroimmun. Both ELISA assays showed comparable performance in identifying patients at risk of strongyloidiasis and in monitoring serological response after ivermectin treatment, supporting their usefulness in the management of patients with CD and suspected strongyloidiasis. Routine serological screening should be considered in this high-risk population.

## 1. Introduction

In recent decades, increasing migration from Latin America to several European countries, including Spain, has led to greater recognition of parasitic diseases endemic to the Americas, such as Chagas disease (CD). In addition, a rise in less prevalent infections, including strongyloidiasis, has been observed [1,2,3,4].

Strongyloidiasis and CD are neglected tropical diseases that share similar epidemiological profiles in Latin America. Both primarily affect rural and suburban areas and are associated with low socioeconomic conditions [5]. Globally, strongyloidiasis and CD are estimated to affect about 386 million and 7 million people, respectively [2,6]. Among people with CD, the reported seroprevalence of *Strongyloides stercoralis* ranges from 10% to 44% [5,7,8].

This high prevalence of strongyloidiasis among those infected with *Trypanosoma cruzi*, potential complications associated with *S. stercoralis* infection, and the benefits of ivermectin treatment provide strong justification for systematic screening in these populations. Direct demonstration of larvae in stool samples provides parasitological confirmation of infection, although its sensitivity is limited in chronic strongyloidiasis [9,10]. Therefore, the limited sensitivity of these methods has driven the commercial availability of various serological assays for detecting antibodies against *S. stercoralis* [11,12]. In addition, the dynamics of the antibody response are influenced by the chronic nature of the infection and the parasite’s autoinfective cycle, which may contribute to distinct serological patterns [13].

Since screening for *S. stercoralis* infection is recommended for people from high-prevalence areas [5]. The aim of this study was to evaluate the agreement between diagnostic performance and utility for monitoring the serological response of two commercial serological assays for strongyloidiasis in patients with CD. These individuals had previously received etiological treatment for *T. cruzi* infection and were routinely followed up because there are no validated biomarkers to confirm a cure for CD.

## 2. Materials and Methods

### 2.1. Study Design and Setting

This was an observational cross-sectional study with a one-year follow-up. Diagnostic evaluation for *S. stercoralis* infection was performed during patients’ visits to the Tropical Medicine Unit of Hospital Clínico Universitario Virgen de la Arrixaca (Murcia, Spain). The study was conducted between 2020 and 2022 at the hospital’s Microbiology Department.

### 2.2. Study Population

We included 55 patients with a prior diagnosis of CD, confirmed using WHO criteria [14]. All were attending the Tropical Medicine Unit for follow-up after etiological therapy for *T. cruzi* infection. Currently, a CD cure cannot be confirmed because there are no validated biomarkers of parasitological clearance. As part of the follow-up, patients were systematically offered a serological screening for *S. stercoralis* infection.

### 2.3. Inclusion and Exclusion Criteria

The inclusion criteria were confirmed diagnosis of CD according to WHO criteria, serological screening for *S. stercoralis* performed during follow-up, and availability of epidemiological and laboratory data at the time of evaluation.

Exclusion criteria included incomplete clinical or laboratory information.

### 2.4. Collected Variables

We recorded demographic and epidemiological variables, including sex, age, country of origin, and length of residence in Spain. Laboratory data included peripheral blood eosinophil counts available at the time of evaluation. Eosinophilia was defined as an eosinophil count > 0.5 × 10^3^ eosinophils per µL.

Total leukocyte counts, neutrophil counts, lymphocyte counts and other complete blood count parameters were not systematically collected in a structured format as part of the original study dataset.

### 2.5. Serological Assays for Strongyloidiasis

We used two commercial ELISA assays to diagnose and follow *S. stercoralis* infection: the Strongyloides IgG ELISA kit (DRG Instruments GmbH, Marburg, Germany) (DRG), which uses *S. stercoralis* larval extract as the antigen, and the Anti-Strongyloides ELISA IgG (Euroimmun Medizinische Labordiagnostika AG, Lübeck, Germany) (Euroimmun), which uses larval extracts from *Strongyloides papillosus* containing antigenic determinants shared with *S. stercoralis* and other members of the genus.

All assays were performed according to the manufacturers’ instructions. The result was considered positive when the index value was >1.1.

For the ELISA-DRG assay, a reactivity index was calculated according to the method described by Bon et al. [15] by dividing the sample optical density (OD) by a fixed reference value of 0.200.

### 2.6. Case Definition Using a Composite Reference Standard

Direct parasitological methods were not systematically performed because the study was conducted as part of the routine follow-up of patients with Chagas disease, in which serological screening for strongyloidiasis was included in clinical care. Consequently, stool samples suitable for parasitological confirmation were not consistently available for all participants.

Since stool samples were not available for direct parasitological confirmation, we used a composite reference standard. A patient was classified as having study-defined strongyloidiasis (infected) if they had positive serology for *S. stercoralis* with >0.5 × 10^3^ eosinophils per µL or positive serology for *S. stercoralis* by both assays.

### 2.7. Strongyloidiasis Treatment and Follow-Up

All patients classified as infected received presumptive ivermectin treatment at a dose of 200 µg/kg/day for 2 consecutive days. Follow-up assessments were conducted at one year and, when available, at two years post-treatment, including both serological assays and eosinophil counts.

A therapeutic response was defined as either seroconversion to negative in any of the assays or a post-treatment/pre-treatment index ratio < 0.6 at one year [9,16].

### 2.8. Ethical Considerations

The study was conducted in accordance with the principles of the Declaration of Helsinki. Ethical approval was obtained from the institutional review board of Hospital Clínico Universitario Virgen de la Arrixaca (2020-1-11-HCUVA).

### 2.9. Statistical Analysis

Statistical analyses were performed using IBM SPSS Statistics version 17.0 (IBM Corp., Armonk, NY, USA). Sensitivity and specificity were calculated from 2 × 2 contingency tables using the composite reference standard. Agreement between the two serological assays was assessed using Cohen’s kappa coefficient [17].

Changes in eosinophil counts and ELISA indices before and after treatment were analyzed using the Wilcoxon signed-rank test. A *p*-value < 0.05 was considered statistically significant.

## 3. Results

### 3.1. Baseline Characteristics of the Study Population

A total of 55 patients previously diagnosed with and treated for CD were evaluated for *S. stercoralis* infection. The year of the first consultation (range: 2007–2015) corresponded to the period immediately following migration to Spain, since Bolivian nationals could enter Spain without a visa until 2007. This variable, therefore, represents the approximate time of arrival and the initiation of clinical follow-up.

All participants were established residents in Spain, although many reported occasional short trips to Bolivia. Accordingly, the variable of years since last travel reflects the number of years since the last potential exposure to *S. stercoralis*. The mean interval since the last trip to Bolivia was 7.7 ± 3.0 years, suggesting predominantly long-standing imported infections with limited opportunity for recent re-exposure.

The mean age was 37.7 ± 9.0 years, and 52.7% were women. Nearly all participants (98.2%) were born in Bolivia. Baseline characteristics are presented in Table 1 and Supplementary Table S1.

A comparison of demographic variables between infected and non-infected patients revealed no significant differences regarding age, sex, region of origin, or years since last travel to Bolivia (Table 1).

### 3.2. Performance of Serological Assays According to the Composite Reference Standard

Using the composite reference standard, overall, 37/55 patients (67.3%) were classified as infected with *S. stercoralis*. Of these, 34 were positive by both ELISA assays, and three were positive only by the Euroimmun assay while also presenting 0.5 × 10^3^ eosinophils per µL.

Agreement between the two assays was substantial, with an overall concordance of 90.9% and a Cohen’s kappa coefficient of 0.80 after recording borderline results as negative. The diagnostic performance of each serological test is shown in Table 2.

### 3.3. Strongyloidiasis Treatment and Follow-Up

Of the 37 patients classified as infected, 30 (81.1%) attended the one-year follow-up visit after ivermectin treatment. The evolution of serological markers and eosinophils showed a significant decline.

• DRG index (mean ± SD): 8.49 ± 3.95 → 2.86 ± 3.48 (*p* < 0.01).
• Euroimmun index (mean ± SD): 6.39 ± 2.24 → 2.44 ± 1.95 (*p* < 0.01).
• Eosinophil count (mean ± SD): 1.16 ± 0.66 → 0.31 ± 0.39 (*p* < 0.01).

**Figure 1:**
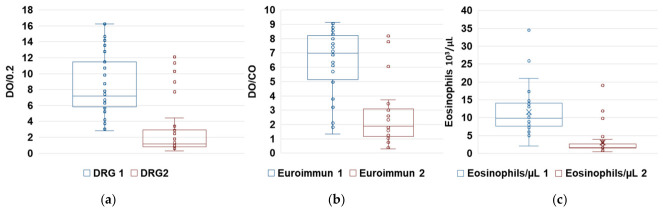
Evolution of markers: (**a**) antibody reactivity by DRG ELISA; (**b**) antibody reactivity by Euroimmun ELISA. (**c**) Eosinophil counts.

These findings indicate a consistent serological response accompanied by a marked reduction in peripheral blood eosinophilia after ivermectin treatment (Figure 1).

Based on predefined response criteria (seroreversion or post-/pre-index ratio < 0.6), the treatment response was:

• DRG ELISA: 25/30 responders (83.3%).
• Euroimmun ELISA: 22/30 responders (73.3%).
• Both assays: 22/30 responders (73.3%).

Detailed results are presented in Supplementary Table S2.

Among the five patients classified as non-responders by both assays (cases 7, 8, 15, 19, 20), three showed a downward trend in serological reactivity and eosinophil counts (cases 7, 8, 20), whereas the remaining two exhibited either a slight increase or persistent elevation across all three markers (Table 3). These patients received a second course of ivermectin treatment. A reduction in antibody levels, as measured by the DRG assay, was observed in four cases, while one patient did not attend the post-treatment follow-up visit (Supplementary Table S3).

Among three patients who were classified as non-responders by the Euroimmun assay (cases 10, 27, and 28) but fulfilled response criteria in the DRG assay (ratio < 0.6), DRG indices at one-year follow-up decreased and approached the assay cut-off, whereas Euroimmun indices showed limited change. In addition, persistent eosinophilia was observed in case 10 (Table 4). This patient received a second course of ivermectin but was subsequently lost to follow-up.

## 4. Discussion

CD and strongyloidiasis are both endemic in South America and are associated with considerable morbidity and mortality. Because most patients chronically infected with *S. stercoralis* remain asymptomatic and share epidemiological characteristics with those affected by CD, screening for strongyloidiasis in individuals infected with *T. cruzi* is strongly recommended [5,18]. In our study, this approach enabled the identification of 37 cases of coinfection over a 2-year period.

The long interval since the last potential exposure observed in our cohort is compatible with the natural history of *S. stercoralis* infection. Unlike most intestinal nematodes, *S. stercoralis* is capable of completing its life cycle within the human host through an internal autoinfective cycle, allowing infection to persist for decades in the absence of reinfection. Chronic infections may therefore remain asymptomatic and undiagnosed for many years, and cases lasting more than 60 years after leaving endemic areas have been documented [19]. This biological characteristic supports the likelihood that most infections identified in our study represented long-standing chronic infections acquired before migration rather than recent exposures.

The association between Chagas disease and strongyloidiasis extends beyond their shared epidemiological distribution. Both infections are highly prevalent in rural and socioeconomically vulnerable regions of Latin America, resulting in frequent coinfection. Several studies have reported a higher prevalence of *S. stercoralis* infection among individuals with *T. cruzi* infection than in the general population from the same endemic areas [5,7]. Furthermore, chronic helminth infections may modulate the host immune response through Th2-polarized and regulatory pathways, potentially influencing the immunological profile associated with chronic Chagas disease [18]. Previous studies have also suggested that strongyloidiasis may be associated with increased detection of *T. cruzi* DNA in peripheral blood, indicating a possible effect on parasite persistence or parasitaemia [8]. These epidemiological and biological interactions make strongyloidiasis a particularly relevant coinfection among patients with Chagas disease and justify implementing targeted screening strategies in this population. Unlike most intestinal protozoa and other common enteric parasites, *S. stercoralis* can establish lifelong infection through autoinoculation and may cause severe hyperinfection syndrome in immunocompromised individuals, making its detection and treatment a public health priority.

Traditionally, the diagnosis of strongyloidiasis has relied on parasitological stool examination. However, its sensitivity remains suboptimal because of the low and intermittent excretion of larvae, often requiring repeated examinations and/or concentration techniques such as Baermann or agar plate culture [9]. In recent decades, several serological methods have been developed and commercialized, reducing costs and improving sensitivity and specificity [20]. Among them, ELISA-based assays are the most widely used and appear to offer the highest diagnostic performance [10].

In our study, the two evaluated commercial ELISA assays showed good agreement and identified a similar group of patients classified as infected according to the study definition, consistent with previous findings [15,21]. These findings support the use of these assays as practical tools for serological screening and follow-up in populations at risk of strongyloidiasis.

An important methodological difference between the evaluated assays is the nature of the antigen used. While the DRG ELISA employs *S. stercoralis* larval extract, the Euroimmun assay uses larval antigens derived from *S. papillosus*. The diagnostic performance of the latter relies on the presence of shared antigenic epitopes among *Strongyloides* species, enabling detection of cross-reactive anti-*Strongyloides* antibodies in patients infected with *S. stercoralis*. Consequently, the Euroimmun assay should be regarded as detecting genus-level rather than strictly species-specific antibody responses. Although this strategy may theoretically increase cross-reactivity with antibodies directed against other helminths, previous studies and our results demonstrate a high degree of concordance between both assays, supporting its usefulness for clinical screening and follow-up. Nevertheless, the absence of species-specific antigens should be considered when interpreting positive serological results, particularly in individuals with potential exposure to multiple helminth infections. This consideration is particularly relevant in our study because serological testing for other helminth infections was not performed.

An important limitation of this study is the absence of independent parasitological confirmation. Stool-based methods such as Baermann, agar plate culture, repeated stool microscopy, or PCR were not systematically performed because samples were not routinely collected within the clinical follow-up from which this cohort was derived. Consequently, infection status was established using a composite reference standard based on serological findings and eosinophilia. Although this approach was intended to identify individuals with a high probability of infection in a real-world clinical setting, it may have introduced a bias, potentially leading to overestimation of sensitivity, specificity, positive predictive value, and negative predictive value. Furthermore, while stool-based techniques could have provided an independent source of diagnostic confirmation, their sensitivity in chronic strongyloidiasis is often limited by the low and intermittent excretion of larvae.

A further limitation is that complete blood count parameters other than absolute eosinophil counts were not systematically collected in a structured format. Therefore, we could not assess total leukocyte counts, neutrophil or lymphocyte counts, leukopenia, leukocytosis, or other hematological abnormalities. Consequently, the interpretation of hematological findings was restricted to the available peripheral blood eosinophil counts.

Therefore, the diagnostic parameters reported in this study should be interpreted with caution and regarded as measures of agreement with the study case definition rather than definitive estimates of diagnostic accuracy.

The use of a composite reference standard may also have resulted in some degree of misclassification. In particular, eosinophilia may occur in allergic disorders or infections caused by other helminths, potentially leading to false-positive classification in a small number of patients. The possibility of misclassification due to cross-reactive antibodies cannot be completely excluded [22,23]. Although the long duration of residence in Spain and the prolonged interval since the last travel to endemic areas make active infection with other helminths less likely [24], some participants reported occasional return visits to Bolivia, and therefore, recent re-exposure to *S. stercoralis* or other helminths cannot be completely ruled out. Furthermore, borderline serological results were classified as negative. Although this conservative approach reduced the likelihood of false-positive classification, it may also have led to underestimation of infection in a small number of individuals. However, in our cohort, this had no substantial impact on the overall interpretation of the study findings.

The timing of post-treatment evaluation is critical for interpreting therapeutic response. Reported cure rates vary widely, from 3.4% at three months to 91.7% at 18 months post-treatment [25]. In our cohort, 80% of patients met the response criteria one year after treatment according to the DRG ELISA and 70% according to the Euroimmun ELISA. These findings suggest that both ELISAs may be useful tools for monitoring treatment response, enabling confirmation of serological cure in most patients after one year while limiting extended follow-up to a smaller subgroup. Similar observations have been reported by Salvador et al. [16], who described a reduction in anti-*Strongyloides* antibody levels in 81% of treated patients at six months post-treatment using a comparable ELISA approach. This suggests that treatment response may be detectable earlier, although later time points may provide more robust confirmation. Nevertheless, a decline in antibody levels should not be interpreted as definitive evidence of a parasitological cure. Because *S. stercoralis* is capable of maintaining infection through its autoinfective cycle, residual infection may persist despite a reduction in antibody titres. Therefore, serological response should be regarded as an indirect marker of treatment success rather than a definitive indicator of parasite eradication.

A small number of patients showed discordant serological responses during follow-up, with response criteria met in one ELISA but not in the other (Table 4). In these three cases, serological response was observed earlier in the DRG assay, while Euroimmun remained positive, possibly reflecting differences in analytical sensitivity and antibody kinetics. As no further follow-up data were available, it was not possible to determine whether these patients would have met response criteria at later time points. These findings highlight the potential value of extended monitoring in selected cases.

Chronic CD is not typically associated with eosinophilia; therefore, its presence should prompt consideration of a concomitant helminth infection [18]. Although the correlation between eosinophilia and seropositivity for *S. stercoralis* is limited, eosinophilia is more frequently observed in infected individuals, and its absence does not exclude the diagnosis of strongyloidiasis [9,26]. In our cohort, 96.7% of patients classified as having study-defined strongyloidiasis presented with eosinophilia, which decreased significantly after treatment (*p* < 0.001), supporting a hematological response to ivermectin.

Despite these limitations, this study provides comparative data on two widely used commercial ELISA assays in a well-characterized cohort of patients with Chagas disease and strongyloidiasis, including longitudinal assessment after ivermectin treatment. Such data remain limited in non-endemic settings and may help guide screening and follow-up strategies in migrant populations.

## 5. Conclusions

Overall, our results support the use of both ELISA assays (DRG and Euroimmun) for serological screening and for monitoring serological response after ivermectin treatment in patients at risk of *S. stercoralis* infection. In our cohort, eosinophilia was present in nearly all patients classified as having study-defined strongyloidiasis, supporting its value as a common accompanying finding, although its absence does not rule out infection.

## Figures and Tables

**Table 1:** Characteristics of participants according to the study-defined strongyloidiasis classification.

	Infected (n = 37)	Uninfected (n = 18)
**Age**		
Median (IQR)	35 (31–44)	36 (31–44.5)
**Sex**		
Female	18	11
Male	19	7
**Country of origin**		
Bolivia	37	17
Argentina	0	1
**Region of origin**		
Cochabamba	23	9
Santa Cruz	8	7
Chuquisaca	4	0
Tarija	2	1
Mendoza	0	1
**Years since last travel**		
Median (IQR)	8 (7–10)	7 (5–9)
**DRG index**		
Median (IQR)	6.96 (5.22–11.50)	0.45 (0.07–0.94)
**Euroimmun index**		
Median (IQR)	6.48 (3.88–8.00)	0.30 (0.15–0.75)

**Table 2:** Estimation of diagnostic parameters of strongyloidiasis tests by binomial distribution analysis.

Test	Infectious Status		Sensitivity ^1^ %	Specificity ^1^ %
	Infected	Uninfected	(95% CI)	(95% CI)
**DRG**				
Positive	34	2	91.9	88.9
Negative	3	16	(78.7–97.2)	(67.2–96.9)
**Euroimmun**				
Positive	37	0	100	100
Negative	0	18	(90.6–100)	(82.4–100)

^1^ Differences in sensitivity and specificity between assays were evaluated using the exact McNemar test; no significant discrepancies were observed (*p* > 0.05). Because the evaluated ELISA assays contributed to the study-defined composite classification, these estimates should not be interpreted as measures of true diagnostic accuracy but rather as agreement with the study case definition.

**Table 3:** Non-responders at one-year follow-up according to both ELISA assays.

Case	DRG1	DRG2	Ratio DRG	Euroimmun1	Euroimmun2	Ratio Euroimmun	EOS1 (10^3^/µL)	EOS2 (10^3^/µL)
7	11.51	8.96	0.8	3.78	3.18	0.8	1.10	0.11
8	11.50	7.72	0.7	4.96	3.73	0.8	1.30	0.19
15	7.42	11.36	1.5	8.57	7.79	0.9	1.47	1.19
19	6.69	12.12	1.8	5.7	8.19	1.4	0.62	0.98
20	6.62	4.41	0.7	1.81	1.58	0.9	3.45	0.33

DRG: DRG ELISA index; Euroimmun: Euroimmun ELISA index; Ratio: post-treatment value divided by pre-treatment value; Eosinophils (10^3^/µL): eosinophil count in peripheral blood. Determination 1 corresponds to pre-treatment, and determination 2 to the 1-year post-treatment follow-up. The response threshold was defined as (post-/pre-treatment ratio < 0.6) in both ELISAs. Eosinophilia > 0.5 × 10^3^/µL.

**Table 4:** Discordant therapeutic response at one-year follow-up.

Case	DRG1	DRG2	Ratio DRG	Euroimmun1	Euroimmun2	Ratio Euroimmun	EOS1 (10^3^/µL)	EOS2 (10^3^/µL)
10	10.70	1.12	0.1	6.48	6.07	0.9	1.06	0.47
27	3.74	1.52	0.4	4.96	3.63	0.7	0.67	0.15
28	3.30	1.03	0.3	1.33	1.03	0.8	0.80	0.19

DRG: DRG ELISA index; Euroimmun: Euroimmun ELISA index; Ratio: post-treatment value divided by pre-treatment value; Eosinophils (10^3^/µL): eosinophil count in peripheral blood. Determination 1 corresponds to pre-treatment, and determination 2 to the 1-year post-treatment follow-up. The response threshold was defined as (post-/pre-treatment ratio < 0.6) in both ELISAs. Eosinophilia > 0.5 × 10^3^/µL.

## Data Availability

The raw data supporting the reported results are in the Supplementary Tables.

## Supplementary Materials

The following supporting information can be downloaded at: https://www.mdpi.com/article/10.3390/pathogens15060627/s1, Supplementary Table S1: Baseline characteristics of individuals included in the study. Supplementary Table S2: The evolution of serological markers and the eosinophil counts. Supplementary Table S3: Changes in anti-*Strongyloides* antibody levels measured by the DRG assay in patients receiving two courses of ivermectin.

## Abbreviations

The following abbreviations are used in this manuscript:

CD	Chagas Disease
WHO	World Health Organization
