# Supplementary material for: Assessment of Two Commercial Serological Assays for the Diagnosis and Post-Treatment Follow-Up of Strongyloidiasis in a Cohort of Patients with Chagas Disease
Source: Pathogens. 2026 Jun 12;15(6):627. doi: 10.3390/pathogens15060627 (PMC13304599; doi:10.3390/pathogens15060627)
Supplement: Supplementary file 1 [file pathogens-15-00627-s001.zip › Supplementary Table S1.pdf]

Supplementary Table S1. Baseline characteristics of individuals included in the study

ID Patient	Sex	Age	Year of first consulta tion	Country of origin	Region of origin	Years since last travel	CD treatment	Year of last visit to endemic region	DRG Index	DRG result	Euroimmun index	Euroimmun result	Case definition
1	H	62	2007	BOLIVIA	SANTA CRUZ	17	BZN	2003	16.27	POS	7.65	POS	Infected
2	M	46	2009	BOLIVIA	CHUQUISACA	6	BZN	2014	16.24	POS	8.55	POS	Infected
3	M	27	2014	BOLIVIA	COCHABAMBA	7	BZN	2013	15.49	POS	6.42	POS	Infected
4	M	34	2009	BOLIVIA	COCHABAMBA	3	BZN	2017	15.25	POS	8.30	POS	Infected
5	M	43	2012	BOLIVIA	COCHABAMBA	8	BZN	2012	14.67	POS	9.13	POS	Infected
6	H	44	2011	BOLIVIA	COCHABAMBA	11	BZN	2009	14.18	POS	2.10	POS	Infected
7	H	35	2007	BOLIVIA	COCHABAMBA	4	BZN	2016	13.56	POS	8.56	POS	Infected
8	H	44	2009	BOLIVIA	COCHABAMBA	3	BZN	2017	12.78	POS	8.30	POS	Infected
9	H	47	2014	BOLIVIA	SANTA CRUZ	10	BZN	2010	11.51	POS	3.78	POS	Infected
10	H	43	2008	BOLIVIA	COCHABAMBA	4	BZN	2016	11.5	POS	7.27	POS	Infected
11	H	31	2009	BOLIVIA	COCHABAMBA	8	BZN	2012	11.5	POS	4.96	POS	Infected
12	M	38	2014	BOLIVIA	COCHABAMBA	10	BZN	2010	11.33	POS	7.90	POS	Infected
13	H	38	2014	BOLIVIA	SANTA CRUZ	10	BZN	2010	10.7	POS	6.48	POS	Infected
14	M	48	2015	BOLIVIA	SANTA CRUZ	10	BZN	2010	9.82	POS	5.80	POS	Infected
15	H	33	2014	BOLIVIA	COCHABAMBA	11	BZN	2009	8.74	POS	6.22	POS	Infected
16	H	53	2009	BOLIVIA	CHUQUISACA	7	BZN	2013	7.79	POS	6.12	POS	Infected
17	M	25	2009	BOLIVIA	COCHABAMBA	2	BZN	2018	7.53	POS	7.51	POS	Infected
18	H	23	2012	BOLIVIA	COCHABAMBA	9	BZN	2011	7.42	POS	8.57	POS	Infected
19	H	31	2015	BOLIVIA	SANTA CRUZ	9	BZN	2011	6.96	POS	7.40	POS	Infected
20	M	28	2014	BOLIVIA	COCHABAMBA	10	BZN	2010	6.95	POS	8.70	POS	Infected
21	H	55	2015	BOLIVIA	COCHABAMBA	8	BZN	2012	6.72	POS	8.78	POS	Infected
22	M	31	2015	BOLIVIA	TARIJA	11	BZN	2009	6.69	POS	5.70	POS	Infected
23	M	29	2014	BOLIVIA	CHUQUISACA	8	BZN	2012	6.62	POS	1.81	POS	Infected
24	M	38	2011	BOLIVIA	COCHABAMBA	8	BZN	2012	6.49	POS	9.06	POS	Infected
25	M	32	2012	BOLIVIA	COCHABAMBA	7	BZN	2013	6.32	POS	8.00	POS	Infected
26	M	33	2011	BOLIVIA	COCHABAMBA	9	BZN	2011	5.69	POS	3.88	POS	Infected
27	M	28	2012	BOLIVIA	SANTA CRUZ	7	BZN	2013	5.69	POS	6.34	POS	Infected
28	H	48	2012	BOLIVIA	COCHABAMBA	9	BZN	2011	5.22	POS	6.87	POS	Infected

29	H	42	2014	BOLIVIA	CHUQUISACA	9	BZN	2011	4.2	POS	7.11	POS	Infected
30	M	31	2008	BOLIVIA	SANTA CRUZ	4	BZN	2016	3.74	POS	4.96	POS	Infected
31	M	34	2013	BOLIVIA	SANTA CRUZ	6	BZN	2014	3.3	POS	1.33	POS	Infected
32	H	36	2014	BOLIVIA	COCHABAMBA	8	BZN	2012	3.02	POS	3.21	POS	Infected
33	M	29	2012	BOLIVIA	COCHABAMBA	9	BZN	2011	2.82	POS	7.87	POS	Infected
34	M	49	2014	BOLIVIA	SANTA CRUZ	11	BZN	2009	2.485	POS	0.15	NEG	Uninfected
35	H	34	2010	BOLIVIA	TARIJA	7	BZN	2013	2.38	POS	0.91	BORDERLINE	Uninfected
36	H	34	2010	BOLIVIA	TARIJA	7	BZN	2013	1.455	POS	1.92	POS	Infected
37	M	30	2012	BOLIVIA	COCHABAMBA	6	BZN	2014	1.035	BORDERLINE	0.89	NEG	Uninfected
38	M	38	2010	BOLIVIA	SANTA CRUZ	4	BZN	2016	1.01	BORDERLINE	0.94	BORDERLINE	Uninfected
39	M	34	2009	BOLIVIA	COCHABAMBA	3	BZN	2017	0.965	NEG	0.15	NEG	Uninfected
40	H	46	2014	BOLIVIA	SANTA CRUZ	13	BZN	2007	0.86	NEG	0.47	NEG	Uninfected
41	M	49	2013	BOLIVIA	COCHABAMBA	8	BZN	2012	0.71	NEG	3.08	POS	Infected
42	H	43	2015	BOLIVIA	COCHABAMBA	12	BZN	2008	0.67	NEG	1.50	POS	Infected
43	M	50	2015	BOLIVIA	COCHABAMBA	10	BZN	2010	0.6	NEG	0.15	NEG	Uninfected
44	M	32	2008	BOLIVIA	COCHABAMBA	2	BZN	2018	0.57	NEG	0.63	NEG	Uninfected
45	M	42	2013	BOLIVIA	COCHABAMBA	7	BZN	2013	0.49	NEG	0.79	NEG	Uninfected
46	M	30	2012	BOLIVIA	COCHABAMBA	6	BZN	2014	0.405	NEG	0.14	NEG	Uninfected
47	M	43	2010	BOLIVIA	SANTA CRUZ	2	BZN	2018	0.28	NEG	0.14	NEG	Uninfected
48	H	45	2014	BOLIVIA	SANTA CRUZ	13	BZN	2007	0.27	NEG	1.21	NEG	Uninfected
49	H	27	2015	BOLIVIA	COCHABAMBA	9	BZN	2011	0.25	NEG	2.09	POS	Infected
50	H	42	2014	BOLIVIA	COCHABAMBA	6	BZN	2014	0.08	NEG	0.21	NEG	Uninfected
51	M	31	2009	BOLIVIA	SANTA CRUZ	5	BZN	2015	0.07	NEG	0.30	NEG	Uninfected
52	H	31	2007	BOLIVIA	SANTA CRUZ	6	BZN	2014	0.06	NEG	0.30	NEG	Uninfected
53	M	55	2013	BOLIVIA	COCHABAMBA	8	BZN	2012	0.06	NEG	0.04	NEG	Uninfected
54	H	26	2012	BOLIVIA	COCHABAMBA	7	NFX	2013	0.02	NEG	0.31	NEG	Uninfected
55	H	26	2015	ARGENTINA	MENDOZA	8	BZN	2012	0.02	NEG	0.12	NEG	Uninfected

BNZ, Benznidazole; NFX, Nifurtimox; POS, positive; NEG, negative.
