# Supplementary material for: Assessment of Two Commercial Serological Assays for the Diagnosis and Post-Treatment Follow-Up of Strongyloidiasis in a Cohort of Patients with Chagas Disease
Source: Pathogens. 2026 Jun 12;15(6):627. doi: 10.3390/pathogens15060627 (PMC13304599; doi:10.3390/pathogens15060627)
Supplement: Supplementary file 1 [file pathogens-15-00627-s001.zip › Supplementary Table S2.pdf]

Supplementary Table S2. The evolution of serological markers and the eosinophil counts

Patient ID	DRG 1	Euroimmun 1	Eosinophils 1 ($10^3/\mu L$)	DRG 2	Euroimmun 2	Eosinophils 2 ($10^3/\mu L$)	RATIO DRG	RATIO EUROIMMUN
1	16.27	7.65	2.59	10.29	2.33	0.39	0.6	0.3
2	16.24	8.55	0.50	3.38	1.8	0.16	0.2	0.2
3	14.67	9.13	1.00	0.65	1.24	0.15	0.0	0.1
4	14.18	2.1	0.90	2.88	1.31	0.15	0.2	0.6
5	13.56	8.56	1.47	0.67	0.31	0.17	0.0	0.0
6	12.78	8.3	1.10	1.78	3.45	0.11	0.1	0.4
7	11.51	3.78	0.21	8.965	3.18	0.05	0.8	0.8
8	11.5	4.96	1.10	7.725	3.73	0.11	0.7	0.8
9	11.5	7.27	1.30	1.065	2.33	0.19	0.1	0.3
10	10.7	6.48	1.06	1.12	6.07	0.47	0.1	0.9
11	9.82	5.8	0.85	2.92	3.14	0.19	0.3	0.5
12	8.74	6.22	1.38	0.62	0.38	0.17	0.1	0.1
13	7.79	6.12	0.95	0.87	0.77	0.25	0.1	0.1
14	7.53	7.51	1.30	0.67	2.6	0.16	0.1	0.3
15	7.42	8.57	1.47	11.36	7.79	1.19	1.5	0.9
16	6.96	7.4	0.89	0.61	0.82	0.08	0.1	0.1
17	6.95	8.7	0.78	2.48	2.67	0.15	0.4	0.3
18	6.72	8.78	1.73	0.93	1.06	0.25	0.1	0.1
19	6.69	5.7	0.62	12.12	8.19	0.98	1.8	1.4
20	6.62	1.81	3.45	4.41	1.58	0.33	0.7	0.9
21	6.49	9.06	1.80	1.28	1.14	1.90	0.2	0.1
22	6.32	8	0.87	1.35	2.51	0.14	0.2	0.3
23	5.69	3.88	0.60	1.03	1.34	0.16	0.2	0.3
24	5.69	6.34	2.10	0.28	0.45	0.24	0.0	0.1
25	5.22	6.87	0.54	1.265	1.94	0.29	0.2	0.3
26	4.2	7.11	0.87	0.96	1.62	0.15	0.2	0.2
27	3.74	4.96	0.67	1.52	3.63	0.15	0.4	0.7
28	3.3	1.33	0.80	1.03	1.03	0.19	0.3	0.8
29	3.02	3.21	0.72	0.82	1.68	0.09	0.3	0.5
30	2.82	7.51	1.10	0.665	3	0.16	0.2	0.4
