# Supplementary material for: Assessment of Two Commercial Serological Assays for the Diagnosis and Post-Treatment Follow-Up of Strongyloidiasis in a Cohort of Patients with Chagas Disease
Source: Pathogens. 2026 Jun 12;15(6):627. doi: 10.3390/pathogens15060627 (PMC13304599; doi:10.3390/pathogens15060627)
Supplement: Supplementary file 1 [file pathogens-15-00627-s001.zip › Supplementary Table S3.pdf]

Supplementary Table S3. Changes in anti-*Strongyloides* antibody levels measured by the DRG assay in patients receiving two courses of ivermectin

Patient ID	DRG 1	DRG 2	DRG 3
7	11.51	8.97	0.38
8	11.50	7.73	2.27
15	7.42	11.36	No sample
19	6.69	12.12	0.44
20	6.62	4.41	1.12
